# Experiences in the design and implementation of phase II trials in CLL

**DOI:** 10.1186/1745-6215-12-S1-A92

**Published:** 2011-12-13

**Authors:** Dena R Cohen, Peter Hillmen, Julia M Brown, Walter M Gregory

**Affiliations:** 1 Clinical Trials Research Unit, University of Leeds, Leeds, UK; 2 Leeds Teaching Hospitals NHS Trust, St James's University Hospital, Leeds, UK

## 

Since 2004, we have developed five phase II trials in Chronic Lymphocytic Leukaemia (CLL), utilising six different statistical methods. Two of the trials have closed to recruitment, two are currently open and a further one is in development. The rationale behind the different designs chosen for each trial will be explained. Difficulties and learning experiences with the implementation, wider understanding and interpretation of the trials will be discussed.

CLL201 used Gehan’s two-stage approach to assess response, and randomised to a control arm which was not included for formal comparison, but to give validity of the study results. Challenges included the timing of the stage I analysis without halting recruitment, and the temptation to formally compare the two arms even though there was not power to do so. The inclusion of the control arm proved to be valuable since the response rates were not as expected.

CLL207 is a single arm trial designed using Bryant and Day’s two-stage design, incorporating toxicity considerations as well as efficacy. The two-stage aspect worked well in this trial due to the short treatment duration and assessment time. However, implementation was difficult due to the definitions of unacceptable toxicity and unacceptability bounds, and the overlap with the role of the Data Monitoring Committee.

ARCTIC and ADMIRE are two large, randomised phase IIb trials, both formally powered to compare responses against a common control arm. One of the trials assesses non-inferiority. Difficulties were experienced in convincing reviewers that these were not underpowered phase III trials. This design was necessary for the non-inferiority question, as it provides an acceptable certainty of finding the treatment inferior in terms of response before proceeding to a much larger trial to assess longer-term endpoints.

COSMIC is a randomised selection design with two experimental arms. The A’Hern one-stage design is used to determine which of the treatments are eligible to be taken forward for further investigation. In the case where both are acceptable, Sargent & Goldberg’s selection criteria will be applied to determine whether to take forward the treatment with better response rate, or to use alternative selection criteria. The sample size was inflated to ensure acceptable power for selecting the best treatment.

